# Analysis on the Effect of Individualized Aerobic Exercise Intervention for Teenagers with Type 2 Diabetes

**DOI:** 10.2174/1874120701509010262

**Published:** 2015-10-05

**Authors:** Zhao Chun-qi

**Affiliations:** Institute of Physical Education, Zhoukou Normal University, Henan, 466001, China

**Keywords:** Individualized aerobic exercise, intervention, teenagers, type 2 diabetes

## Abstract

This study investigates the intervention effect of individualized aerobic exercise on type 2 diabetes in teenagers. 60 cases of teenager were selected with type 2 diabetes in Zhoukou Hospital of Traditional Medicine from February 2013 to February 2014 as the research object. All enrolled patients’ maximal oxygen and blood glucose fluctuation was tested, followed by individualized aerobic exercise intervention. After 6 months of intervention, the changes in patients’ indexes were compared and the effects of individualized aerobic exercise intervention were evaluated. After the intervention, the patients’ plasma triglyceride and cholesterol content were found significantly lowered than before (P<0.05). However, there’s no significant difference between high and low-density lipoprotein (P>0.05). Moreover, the patients’ insulin and C-peptide levels were found significantly higher than those before intervention (P<0.05); before intervention, their blood glucose and glycated hemoglobin level were higher than normal, after intervention, they were weakened, but there’s no significant difference (P>0.05). The maximal oxygen uptake and different intensity of metabolic equivalents were higher than before, but there’s no significant difference (P>0.05). For teenagers with type 2 diabetes, the implementation of individualized aerobic exercise intervention could effectively improve the patients’ lipid metabolism and cardio-pulmonary function, and effectively promote the insulin and C-peptide secretion, which provides scientific basis for effective control of blood glucose.

## 1. Introduction

Type 2 diabetes is caused by genetic and environmental factors, and the main clinical symptoms are chronic blood glucose rise and protein and lipid metabolism disorders and so on [1]. In recent years, the acceleration in the pace of life and changes in diet structure resulted in the continued rising of obese adolescents rate. In this situation, the incidence caused by obesity in teenagers with type 2 diabetes also increased year by year. In order to improve the living quality of teenagers with type 2 diabetes, it is very important to take effective intervention method. Effective clinical studies have shown that the effect of individualized aerobic exercise intervention is significant in patients with type 2 diabetes. As a result, the effect of aerobic exercise intervention on teenagers with type 2 diabetes is discussed in this paper. The report is as follows:

## 2. Material and methods

### 2.1. General Data

The 60 teenagers chosen in the group are all in line with the diagnosis of type 2 diabetes proposed by American Diabetes Association in 1997 [2]. Among them, there are 26 males and 34 females; aged from 11 to 19, the average age is (16.8±1.2). The course of disease was from 3 months to 3 years, and the average year is (1.8±0.2). 38 of them have oral metformin, and 14 teenagers use insulin, while 8 of them didn’t use hypoglycemic drugs. Before the experiment, the fasting blood glucose and glycosylated hemoglobin were respectively (7.23±1.02) mmol/L、and (6.74±0.49)%.

### 2.2. Methods

#### 2.2.1. Experimental Scheme

(1) Step 1: take a close examination of the selected patients, including their past medical history, medication, exercise and complications, *etc.*, excluding the patients with significant blood glucose fluctuation, acute complications, and patients with severe kidney, heart, hypertension and other diseases. Moreover, check their cardiac and pulmonary function to exclude contraindications to exercise testing defined by ACSM. All selected patients need to sign the “informed consent”, and it should be assisted by their family. (2) Step 2: test the maximal oxygen uptake and the effect of different hypoglycemic intensity. The test should be finished in 4 times, and each interval time is 2d. (3) Step 3: test the hypoglycemic effect at different time, including fasting and postprandial 2 hours blood glucose. In the testing process, the experiment should be based on the patients’ characteristics of glucose, about 2 to 5 times, and each time interval is 2d. (4) Step 4: formulate individualized exercise program for each patient based on the above test results, cover the way, intensity, time and frequency of exercises, *etc.*, in addition, basic items should also be paid attention to in the process. (5) Step 5: test the patients’ body composition before the early period of aerobic exercise intervention and take 3 pipes venous blood to provide a scientific basis for the test of blood glucose and lipid. (6) Step 6: after 6 months exercise intervention, test the above related indexes repeatedly and the maximum oxygen uptake again, and then finally evaluate the effects of intervention.

#### 2.2.2. Intervention Method for Individualized Aerobic Exercise

Individualized aerobic exercise intervention includes many contents, such as: (1) Choose jogging of brisk walking in aerobic exercise. (2) The intensity should be maintained at a moderate level, *i.e.*, 50% VO_2_R [3-4]. (3) Exercise time should be maintained at 15 to 30 minutes; moreover, the warming-up and cooling-down time is 5 minutes. (4) Individualized starting exercise time shall be chosen at 25 to 45 minutes before the peak time based on the peak time of blood glucose and drug effects and combined with individual test results. (5) 3 to 5 times aerobic exercise per week. (6) During movement, the following basic items should be noticed: exercise should be prohibited in fasting condition; it is better to have a partner; carry candy with you to avoid hypoglycemia occurrence. Meanwhile, choose loose sports pants and suitable shoes. In addition, we need to monitor the relevant indicators, such as blood pressure, blood glucose, heart rate and complications, *etc.* [5].

### 2.3. Judgment Standard

During the experiment, the measurement of peripheral blood glucose is required. In this respect, Luokang Active Blood Glucose Meter is used. For the measurement of blood glucose and lipid before and after the intervention, for this group Holland Rittal SELECTRA-EPLUS Automatic Biochemical Analyzer was used. The specific procedure is provided in the instructions. The balanced food intake is needed in the testing process. The glycemic index covers cholesterol, triglycerides and HDL cholesterol. With the help of American Luo CobaSe 411 Electrochemical Luminescence Meter, the insulin and C peptide levels can be tested. After the quality control is finished, the test of routine sample can begin.

### 2.4. Statistical Analysis

All statistical data is analyzed by SPSS 13.O, and it is expressed by mean ± standard deviation, and data count is done by using frequency and percent (%). For categorical data, used x2 test, measurement data and for the comparison between groups, used t-test. The difference is statistically significant only when P < 0.05.

## 3. Results

### 3.1. Comparison of Patients’ Blood Lipid Before and After the Intervention

After the intervention, the patients’ plasma triglyceride and cholesterol contents are significantly lowered than before (P<0.05); there’s no significant difference between high and low-density lipoprotein (P>0.05). See Table **1**.

### 3.2. Comparison of Blood Glucose Before and After Intervention

After the intervention, the patients’ insulin and C-peptide levels are found to be significantly higher than those before intervention (P<0.05); before intervention, their blood glucose and glycosylated hemoglobin (GHB) levels are higher than normal, after intervention, they are weakened, but there’s no significant difference (P>0.05). See Table **2**:

### 3.3. Comparison of the Maximal Oxygen Uptake and Different Intensity of Metabolic Equivalents

The maximal oxygen uptake (VO_2max_) and different intensity of metabolic equivalents are higher than before, but there’s no significant difference (P>0.05). See Table **3**:

## 4. Discussion

According to the *Chinese Guidelines on Prevention and Treatment of Dyslipidemia* in adults, the patients’ triglycerides and cholesterol were at the edge of rise before intervention [6-8]. At the same time, HDL cholesterol was in normal range. After 6 months of intervention, their triglyceride and cholesterol contents decreased significantly. This is mainly because the patients’ lipid mobilization was accelerated in motion. In quite a few situations, free fatty acid concentration based on blood increased, thus providing the required energy substance for liver and muscle. In addition, the skeletal muscle contains glycerol-3-lipid which regulates lipid metabolism. Whereas high density lipoprotein cholesterol increased during intervention, low density lipoprotein cholesterol decreased, but there is no significant difference before and after the intervention (P>0.05). The reason is that: before intervention, the above indicators in these patients are in normal range; thus, the movement has a significant role in their regulation. These patients’ blood glucose is slightly higher than normal. The control of glycosylated hemoglobin is obvious. The two indexes showed no obvious change before and after intervention probably because of two main reasons: first, the patients’ condition is not severe; second, their condition is quite stable. Insulin and C-peptide were improved after intervention, which fully reveals that the effect of individualized aerobic exercise intervention on teenagers with type 2 diabetes is remarkable, and it can effectively improve their islet cell function.

The metabolic equivalent represents the intensity of exercise. It is the common index in exercise intensity. According to *ACSM’s Guidelines for Exercise Testing and Prescription* [9-12], exercise intensity can be divided into 3 grades: 3.2 ~ 5.3MET for low intensity, 5.4 ~ 7.5 for moderate intensity, 7.6 ~ 10.2 for greater intensity. Among 60 patients, moderate and greater intensity are lower than the above standard, and low intensity is close to the standard value, which shows that the patients’ heart and lung function is not ideal before intervention. After 6 months intervention, the low and moderate intensity are close to the standard, and greater intensity is close to the low standard limit. In addition, the maximum oxygen uptake (*i.e.* absolute and relative value) is not up to normal adult standard. After 6 months intervention, there is an increasing trend, meanwhile, the corresponding metabolic equivalent also showed increasing trend. Although there’s no significant difference between two groups of data (P>0.05), we can draw the following conclusion: individualized aerobic exercise can improve the patients’ heart and lung function.

To sum up, for teenagers with type 2 diabetes, the effect of individualized aerobic exercise is significant; it can effectively improve the patients’ lipid metabolism and function of their heart and lung. Meanwhile, it can promote the insulin and C peptide secretion, so that we can provide scientific basis for further effective control of blood glucose.

## Figures and Tables

**Table 1:** Comparison of patients’ blood lipid (X±S,mmol/L) (n=60).

**Group**	**Glycerin triglyceride**	**Cholesterol**	**High-density lipoprotein**	**Low-density lipoprotein**
before intervention	1.98±1.41	5.16±0.48	1.46±0.32	3.65±0.84
after intervention	1.24±1.32	*4.10±0.32	1.48±0.29	3.59±0.86
t	5.361	5.147	1.259	2.126

Note: compared with “before intervention”, *P<0.05.

**Table 2:** Comparison of patients’ blood glucose (X±S, n=60).

**Group**	**Blood glucose (mmol/L)**	**GHB (%)**	**Insulin (μIU/ml)**	**C-peptide (ng/ml)**
before intervention	7.23±1.02	6.76±0.49	6.10±3.26	3.11±1.16
after intervention	6.88±0.92	6.60±0.38	*7.99±3.17	4.33±1.28
t	1.247	1.258	5.257	5.149

Note: compared with “before intervention, *P<0.05.

**Table 3:** Comparison of the maximal oxygen uptake and different intensity of metabolic equivalents (X±S, n=60).

**VO2max**			**Intensity of Metabolic Equivalents**		
	**absolute value (L/min)**	**relative value (ml/kg/min)**	**low intensity**	**moderate intensity**	**high intensity**
before intervention	2.15±0.58	32.05±6.85	3.46±0.49	5.09±0.67	6.72±0.68
after intervention	2.31±0.56	36.02±7.58	*3.78±0.62	5.62±0.73	7.51±0.86
t	1.257	1.568	1.267	2.358	2.317

Note: compared with “before intervention”, *P>0.05.
